# Supplementary material for: The misperception of Asian subgroup representation in STEM
Source: Commun Psychol. 2026 Jan 3;4:21. doi: 10.1038/s44271-025-00389-1 (PMC12873391; doi:10.1038/s44271-025-00389-1)
Supplement: Supplementary file 2 — Supplementary Information for 'The Misperception of Asian Subgroup Representation in STEM' [file 44271_2025_389_MOESM2_ESM.pdf]

Supplementary Information

Supplementary Note 1

For each study, we conducted separate one-way repeated measures Analysis of Variances (ANOVAs) to test if there was a significant main effect of Asian subgroup (Chinese, Japanese, Korean, Indian, Filipino, and Vietnamese) on perceived Asian typicality and perceived status. We conducted Bonferroni-adjusted post hoc comparisons if there were significant main effects. Descriptive statistics and complete pairwise comparison results for perceived Asian typicality are in Table S1, and perceived status are in Table S2.

Perceived Asian Typicality

In Study 1, there was a significant main effect of Asian subgroup on perceived Asian typicality, $F(5,3905) = 144.93$, $p<.001$, $\eta_p^2 = 0.157$. The Chinese subgroup was rated as more typical of the Asian American category than the other Asian subgroups, p's <.001. The Japanese subgroup was rated as more typical of the Asian American category than the Indian, Filipino, and Vietnamese subgroups, p's <.001. The Korean subgroup was rated as more typical of the Asian American category than the Indian, Filipino, and Vietnamese subgroups, p's<.001. Finally, the Vietnamese subgroup was rated as more typical of the Asian American category than both the Indian and Filipino subgroups, $p<.001$.

In Study 2, there was a significant main effect of Asian subgroup on perceived Asian typicality, $F(5,980) = 32.86$, $p<.001$, $\eta_p^2 = 0.151$. The Chinese subgroup was rated as more typical of the Asian American category than the other Asian subgroups, p's <.001. The Korean subgroup was rated as more typical of the Asian American category than the Indian, Filipino, and Vietnamese subgroups, p's$\leq$.005. The Japanese subgroup was rated as more typical of the Asian

American category than the Filipino subgroup, $p<.001$. Finally, the Vietnamese subgroup was rated as more typical of the Asian American category than the Filipino subgroup, $p = .011$.

Table S1. Descriptive statistics and Bonferroni-adjusted post hoc comparisons for perceived Asian typicality results for Studies 1 – 2.

**p<.003 (Bonferroni-adjusted p-value)*

Study	Asian subgroup	Mean (SD)	Pairwise Comparisons: Mean Differences					
			Asian Subgroup					
			Chinese	Japanese	Korean	Indian	Filipino	Vietnamese
1	Chinese	5.69 (1.32)						
	Japanese	5.02 (1.51)	0.66*					
	Korean	5.06 (1.44)	0.63*	-0.03				
	Indian	4.32 (1.68)	1.37*	0.71*	0.74*			
	Filipino	4.41 (1.46)	1.28*	0.61*	0.64*	-0.10		
	Vietnamese	4.64 (1.47)	1.05*	0.39*	0.42*	-0.32*	-0.23*	
2	Chinese	5.62 (1.17)						
	Japanese	4.92 (1.37)	0.70*					
	Korean	5.02 (1.29)	0.60*	-0.10				
	Indian	4.49 (1.55)	1.13*	0.44	0.53*			
	Filipino	4.28 (1.31)	1.34*	0.65*	0.74*	0.21		
	Vietnamese	4.61 (1.36)	1.01*	0.32	0.41*	-0.12	-0.33*	

Perceived Status

In Study 1, there was a significant main effect of Asian subgroup on perceived status, $F(5,3890) = 333.71$, $p<.001$, $\eta_p^2 = 0.300$. The Japanese subgroup was perceived as having higher status than the Korean, Indian, Filipino, and Vietnamese subgroups, p's<.001. The Chinese subgroup was perceived as having higher status than the Korean, Indian, Filipino, and Vietnamese subgroups, p's<.001. The Korean subgroup was perceived as having higher status than the Indian, Filipino, and Vietnamese subgroups, $p\text{'s} \leq .003$. Finally, the Indian subgroup was perceived as having higher status than the Filipino and Vietnamese subgroups, p's<.001.

In Study 2, there was a significant main effect of Asian subgroup on perceived status, $F(5,980) = 99.32$, $p<.001$, $\eta_p^2 = 0.336$. The Japanese subgroup was perceived as having higher status than the Chinese, Korean, Indian, Filipino, and Vietnamese subgroups, p's <.001. The

Chinese subgroup was perceived as having higher status than the Filipino and Vietnamese subgroups, *p*'s <.001. The Korean subgroup was perceived as having higher status than the Filipino and Vietnamese subgroups, *p*'s <.001. Finally, the Indian subgroup was perceived as having higher status than the Filipino and Vietnamese subgroups, *p*'s <.001

Table S2. Descriptive statistics and Bonferroni-adjusted post hoc comparisons for perceived status results for Studies 1 – 2.

**p<.003 (Bonferroni-adjusted p-value)*

Study	Asian subgroup	Mean (SD)	Pairwise Comparisons: Mean Differences					
			Asian Subgroup					
			Chinese	Japanese	Korean	Indian	Filipino	Vietnamese
1	Chinese	6.95 (1.58)						
	Japanese	7.08 (1.52)	-0.13					
	Korean	6.63 (1.59)	0.33*	0.45*				
	Indian	6.36 (1.82)	0.60*	0.73*	0.27*			
	Filipino	5.18 (1.56)	1.78*	1.91*	1.45*	1.18*		
	Vietnamese	5.28 (1.62)	1.67*	1.80*	1.34*	1.07*	-0.11	
2	Chinese	6.80 (1.48)						
	Japanese	7.30 (1.28)	-0.51*					
	Korean	6.80 (1.46)	-0.01	0.50*				
	Indian	6.64 (1.67)	0.15	0.66*	-0.16			
	Filipino	5.21 (1.44)	1.58*	2.09*	1.59*	1.43*		
	Vietnamese	5.32 (1.53)	1.47*	1.98*	1.48*	1.32*	-0.11	

Supplementary Note 2

In Study 1, we examined whether there were any participant race differences in STEM estimates by conducting a 5 (participant race: Asian, Black, Latinx, Pacific Islander, White) x 6 (Asian subgroup: Chinese, Japanese, Korean, Indian, Filipino, Vietnamese) mixed-model ANOVA on STEM estimate differences (i.e., participants' STEM estimate - actual STEM percentage) with Asian subgroup as a repeated-measure factor.

There was not a significant main effect of participant race, $F(1,762) = 1.61$, $p = .169$, $\eta_p^2 = 0.008$. However, there was a significant main effect of Asian subgroup $F(6,4572) = 1351.11$, $p<.001$, $\eta_p^2 = 0.639$, and a significant interaction, $F(24, 4572) = 6.66$, $p<.001$, $\eta_p^2 = 0.034$. We conducted Bonferroni-adjusted post hoc comparisons to examine mean differences between participant races within Asian subgroup. See Table S3 for complete pairwise comparisons. Asian participants were significantly more accurate in their STEM estimates for the Japanese subgroup than Black, Latinx, Pacific Islander, and White participants, *p*'s <.001. Asian participants were significantly more accurate in their STEM estimates for the Indian subgroup than the Black, Latinx, and White participants, *p*'s<.001. Finally, Asian participants were significantly more accurate in their STEM estimates for the Filipino subgroup than the Black participants, $p = .003$.

We additionally conducted a series of one-sample t-tests examining participants' STEM estimates to the actual data from the U.S. census by participant race and subgroup (see Table S4). We still found that participants underestimated Chinese and Indian subgroup representation in STEM and overestimated Japanese, Korean, Filipino, and Vietnamese subgroup representation in STEM, regardless of participant race.

Table S3. Descriptive statistics and Bonferroni-adjusted posthoc comparisons assessing participant race differences in STEM estimates.

*Descriptive statistics for the interaction between Asian subgroup and participant race on STEM estimate difference scores (i.e., participants' STEM estimate – actual STEM percentage) and Bonferroni-adjusted post hoc comparison results for mean differences between participant races within Asian subgroup. *p<.005 (Bonferroni-adjusted p-value)*

Subgroup	Participant Race	Mean (SD)	Pairwise Comparisons: Mean Differences Participant Race				
			Asian	Black	Latinx	Pacific Islander	White
Chinese	Asian	-4.10 (9.53)					
	Black	-6.92 (12.40)	2.813				
	Latinx	-6.10 (11.23)	1.993	-0.82			
	Pacific Islander	-6.03 (11.45)	1.924	-0.89	-0.07		
	White	-6.09 (13.29)	1.991	-0.82	-0.002	0.07	
Japanese	Asian	8.31 (5.86)					
	Black	13.61 (7.84)	-5.30*				
	Latinx	13.60 (9.73)	-5.30*	0.01			
	Pacific Islander	14.39 (8.94)	-6.09*	-0.78	-0.79		
	White	14.76 (8.90)	-6.45*	-1.15	-1.15	-0.36	
Korean	Asian	8.19 (6.07)					
	Black	9.18 (6.26)	-0.99				
	Latinx	9.49 (7.24)	-1.30	-0.31			
	Pacific Islander	9.04 (5.83)	-0.86	0.14	0.44		
	White	9.56 (6.88)	-1.37	-0.38	-0.07	-0.52	
Indian	Asian	-22.59 (11.24)					
	Black	-30.97 (11.98)	8.38*				
	Latinx	-28.77 (13.26)	6.18*	-2.20			
	Pacific Islander	-27.08 (15.26)	4.50	-3.88	-1.68		
	White	-29.54 (13.85)	6.94*	-1.44	0.76	2.45	
Filipino	Asian	5.18 (5.38)					
	Black	7.58 (6.82)	-2.40*				
	Latinx	6.62 (7.11)	-1.44	0.96			
	Pacific Islander	5.92 (4.71)	-0.73	1.67	0.70		
	White	6.20 (6.68)	-1.02	1.38	0.42	-0.29	
Vietnamese	Asian	5.75 (5.63)					
	Black	6.82 (6.70)	-1.07				
	Latinx	5.45 (5.63)	0.30	1.37			
	Pacific Islander	5.42 (4.56)	0.33	1.40	0.03		
	White	5.16 (5.02)	0.59	1.66	0.29	0.26	
Other	Asian	-0.78 (7.13)					
	Black	0.28 (9.70)	-1.06				
	Latinx	-0.30 (8.86)	-0.48	0.58			
	Pacific Islander	-1.32 (7.48)	0.55	1.60	1.03		
	White	0.11 (9.00)	-0.89	0.17	-0.41	-1.43	

Table S4. Descriptive statistics and t-test results assessing participant race differences in STEM estimates.

*Descriptive statistics and individual one-sample t-test results comparing participants' STEM to actual provided by the U.S. Census separated by participant race for Study 1 (N = 784) by Asian subgroup. *p<.05*

Subgroup	Participant Race	*n*	STEM Estimates		
			Actual Value	Mean (SD)	t-value
Chinese	Asian	193	32	27.85 (9.52)	-6.05*
	Black	195	32	24.99 (12.96)	-7.55*
	Latinx	140	32	25.94 (11.26)	-6.37*
	Pacific Islander	73	32	25.67 (11.46)	-4.72*
	White	197	32	25.76 (13.27)	-6.60*
Japanese	Asian	193	2	10.41 (6.02)	19.42*
	Black	195	2	15.84 (8.63)	22.39*
	Latinx	140	2	15.86 (9.92)	16.52*
	Pacific Islander	73	2	16.63 (8.97)	13.94*
	White	197	2	16.78 (8.95)	23.17*
Korean	Asian	193	3	11.23 (6.09)	18.79*
	Black	195	3	12.68 (7.95)	17.00*
	Latinx	140	3	12.36 (7.21)	15.36*
	Pacific Islander	73	3	11.79 (5.95)	12.63*
	White	197	3	12.50 (6.95)	19.18*
Indian	Asian	193	33	27.37 (11.23)	-28.00*
	Black	195	33	19.06 (12.01)	-35.97*
	Latinx	140	33	21.09 (13.21)	-25.89*
	Pacific Islander	73	33	22.37 (15.40)	-15.33*
	White	197	33	20.28 (13.76)	-30.32*
Filipino	Asian	193	2	7.25 (5.44)	13.40*
	Black	195	2	9.82 (6.85)	15.93*
	Latinx	140	2	8.71 (7.08)	11.22*
	Pacific Islander	73	2	7.85 (4.72)	10.58*
	White	197	2	8.11 (6.61)	12.98*
Vietnamese	Asian	193	2	7.82 (5.71)	14.17*
	Black	195	2	9.24 (7.03)	14.39*
	Latinx	140	2	7.50 (5.63)	11.57*
	Pacific Islander	73	2	7.30 (4.56)	9.93*
	White	197	2	7.10 (4.98)	14.37*
Other	Asian	193	9	8.29 (7.16)	-1.39
	Black	195	9	10.44 (12.41)	1.62
	Latinx	140	9	8.61 (8.72)	-0.52
	Pacific Islander	73	9	7.55 (7.42)	-1.67
	White	197	9	8.93 (8.92)	-0.10

Supplementary Note 3

In Study 3, we modified how we asked participants to provide their STEM estimates. Specifically, we changed Americans' reference point when making estimations from the U.S. Asian population to the entire U.S. population. We also manipulated how participants made their STEM estimations by either entering their responses (i.e., the open-ended condition) or selecting their responses from one of the provided categories (i.e., the closed-ended condition). We expected that participants might be more accurate in the closed-ended condition by creating anchoring effects[1,2]. Given the differences in how STEM estimations were measured, we report the analysis for each condition separately.

In the open-ended condition, we modified our STEM estimation item by presenting participants with the following: "Within the United States, a career in the Science, Technology, Engineering, and Mathematics (STEM) fields often requires an advanced degree (e.g., MA, MS, Ph.D.). If you had a random sample of 100 Americans with advanced STEM degrees, how many would be from each of the categories below: White, Black, Chinese, Indian, Japanese, Korean, Filipino, and Vietnamese?" We asked participants to provide STEM estimates for the Black and White racial groups to help participants think about the entire U.S. population rather than just the U.S. Asian American population. Participants were asked to enter their responses, and we conducted one-sample t-tests comparing participants' average STEM estimates to the actual percentages provided by the U.S. Census[3]. Inconsistent with previous studies, participants estimated $M = 15.59$ ($SD = 9.30$) Chinese Americans out of 100 Americans with an advanced STEM degree which is more than the actual percentage (11.1%), $t(225) = 7.27$, $p<.001$, $d = 0.483$. However, consistent with previous studies, participants estimated $M = 8.99$ ($SD = 7.67$) Japanese Americans and $M = 7.68$ ($SD = 6.27$) Korean individuals out of 100 Americans which

are all more than the actual percentage of Japanese (0.5%), $t(225) = 16.64$, $p<.001$, $d = 1.11$ and Korean individuals who have advanced STEM degrees (1.1%), $t(225) = 15.79$, $p<.001$, $d = 1.05$. Additionally, participants estimated $M = 14.06$ ($SD = 9.50$) Indian Americans out of 100 Americans which is more than the actual percentage (17.4%), $t(225) = -5.29$, $p<.001$, $d = -0.352$. Participants estimated $M = 5.55$ ($SD = 5.29$) Filipino and $M = 5.49$ ($SD = 5.69$) Vietnamese individuals which are more than the actual percentage of Filipino (0.6%), $t(225) = 14.07$, $p<.001$, $d = 0.936$; and Vietnamese individuals (0.8%), $t(225) = 12.38$, $p<.001$, $d = 0.823$, represented in STEM. Finally, participants also underestimated White STEM representation ($M = 35.28$, $SD = 17.57$; actual = 56.0%), $t(225) = -17.73$, $p<.001$, $d = -1.18$, and overestimated Black STEM representation ($M = 10.34$, $SD = 7.27$; actual = 7.3%), $t(225) = 6.29$, $p<.001$, $d = 0.418$.

In the closed-ended condition, we presented participants with the following: "Within the United States, a career in the Science, Technology, Engineering, and Mathematics (STEM) fields often requires an advanced degree (e.g., MA, MS, Ph.D.). What percentage of Americans from the following categories below have an advanced STEM degree: White, Black, Chinese, Indian, Japanese, Korean, Filipino, and Vietnamese?" Participants were presented with 12 options: "0-0.9%", "1-1.9%, "2-2.9%", "3-3.9%", "4-4.9%", "5-5.9%", "6-6.9%", "7-7.9%", "8-8.9%", "9-9.9%", "10%", and "greater than 10%". We conducted separate χ^2 analyses for each subgroup to determine if the number of observed participants who selected one of the 12 categories significantly differed from the expected equal distribution. We followed up significant χ^2 results by examining the residuals to determine which most frequently selected category deviated the most from the expected. See Table S5. Participants were more likely to select "greater than 10%" than the other categories for the Chinese subgroup, $\chi^2(11) = 87.59$, $p<.001$, which is consistent with the actual percentage (11.1%). Participants were more likely to select "greater than 10%"

for the Japanese subgroup, $\chi^2(11) = 48.15$, $p<.001$, and “4-4.9%” for Korean Americans, $\chi^2(11) = 30.39$, $p<.001$, both selections are greater than their actual percentages (0.5% and 1.1%). Additionally, participants were more likely to select “greater than 10%” for the Indian subgroup, $\chi^2(11) = 61.37$, $p<.001$, which is consistent with the actual percentage (17.4%). Participants were more likely to select “2-2.9%” for Filipino Americans, $\chi^2(11) = 76.81$, $p<.001$ and “1-1.9%” for Vietnamese Americans, $\chi^2(10) = 48.69$, $p<.001$, both selections are greater than the actual percentages (0.6%, and 0.8%). (Note: the degrees of freedom for Vietnamese Americans is 10 compared to 11 because zero participants selected “greater than 10%”.) Finally, participants were more likely to select “greater than 10%” for White Americans, $\chi^2(11) = 130.09$, $p<.001$, which is consistent with the actual percentage (56%), and more likely to select “2-2.9%” for Black Americans, $\chi^2(11) = 112.33$, $p<.001$, which is less than the actual percentage (7.3%).

Table S5. χ^2 analysis Results

Results for the χ^2 analysis for STEM estimates in Study 3: closed-ended condition.

Subgroup (*Actual*)	χ^2 Results	Category	Observed *N*	Expected *N*	Residual
Chinese *11.1%*	χ^2 (11) = 87.59, p<.001	0 - 0.9%	2	18.9	-16.9
		1 - 1.9%	10	18.9	-8.9
		2 - 2.9%	11	18.9	-7.9
		3 - 3.9%	12	18.9	-6.9
		4 - 4.9%	25	18.9	6.1
		5 - 5.9%	25	18.9	6.1
		6 - 6.9%	16	18.9	-2.9
		7 - 7.9%	21	18.9	2.1
		8 - 8.9%	17	18.9	-1.9
		9 - 9.9%	13	18.9	-5.9
		10%	24	18.9	5.1
		greater than 10%	51	18.9	32.1
Japanese *0.5%*	χ^2 (11) = 48.15, p<.001	0 - 0.9%	1	18.9	-17.9
		1 - 1.9%	13	18.9	-5.9
		2 - 2.9%	15	18.9	-3.9
		3 - 3.9%	20	18.9	1.1
		4 - 4.9%	26	18.9	7.1
		5 - 5.9%	28	18.9	9.1
		6 - 6.9%	17	18.9	-1.9
		7 - 7.9%	18	18.9	-0.9
		8 - 8.9%	24	18.9	5.1
		9 - 9.9%	14	18.9	-4.9
		10%	14	18.9	-4.9
		greater than 10%	37	18.9	18.1
Korean *1.1%*	χ^2 (11) = 30.92, p<.001	0 - 0.9%	5	18.9	-13.9
		1 - 1.9%	15	18.9	-3.9
		2 - 2.9%	20	18.9	1.1
		3 - 3.9%	20	18.9	1.1
		4 - 4.9%	32	18.9	13.1
		5 - 5.9%	21	18.9	2.1
		6 - 6.9%	20	18.9	1.1
		7 - 7.9%	18	18.9	-0.9
		8 - 8.9%	23	18.9	4.1
		9 - 9.9%	14	18.9	-4.9
		10%	11	18.9	-7.9
		greater than 10%	28	18.9	9.1

Table S5 (cont.)

Subgroup (*Actual)*	χ^2 Results	Category	Observed *N*	Expected *N*	Residual
Indian *17.4%*	χ^2 (11) = 61.37, p<.001	0 - 0.9%	8	18.9	-10.9
		1 - 1.9%	10	18.9	-8.9
		2 - 2.9%	17	18.9	-1.9
		3 - 3.9%	20	18.9	1.1
		4 - 4.9%	25	18.9	6.1
		5 - 5.9%	19	18.9	0.1
		6 - 6.9%	19	18.9	0.1
		7 - 7.9%	13	18.9	-5.9
		8 - 8.9%	18	18.9	-0.9
		9 - 9.9%	13	18.9	-5.9
		10%	17	18.9	-1.9
		greater than 10%	48	18.9	29.1
Filipino *0.6%*	χ^2 (11) = 76.81, p<.001	0 - 0.9%	15	18.9	-3.9
		1 - 1.9%	31	18.9	12.1
		2 - 2.9%	40	18.9	21.1
		3 - 3.9%	37	18.9	18.1
		4 - 4.9%	25	18.9	6.1
		5 - 5.9%	15	18.9	-3.9
		6 - 6.9%	17	18.9	-1.9
		7 - 7.9%	8	18.9	-10.9
		8 - 8.9%	9	18.9	-9.9
		9 - 9.9%	8	18.9	-10.9
		10%	12	18.9	-6.9
		greater than 10%	10	18.9	-8.9
Vietnamese *0.8%*	χ^2 (10) = 48.68, p<.001	0 - 0.9%	11	20.6	-9.6
		1 - 1.9%	41	20.6	20.4
		2 - 2.9%	30	20.6	9.4
		3 - 3.9%	32	20.6	11.4
		4 - 4.9%	20	20.6	-0.6
		5 - 5.9%	19	20.6	-1.6
		6 - 6.9%	16	20.6	-4.6
		7 - 7.9%	23	20.6	2.4
		8 - 8.9%	14	20.6	-6.6
		9 - 9.9%	10	20.6	-10.6
		10%	11	20.6	-9.6
		greater than 10%	0	-	-

Table S5 (cont.)

Subgroup (*Actual)*	χ^2 Results	Category	Observed *N*	Expected *N*	Residual
Black	χ^2 (11) = 112.33, *p*<.001	0 - 0.9%	19	18.9	0.1
7.3%		1 - 1.9%	36	18.9	17.1
		2 - 2.9%	47	18.9	28.1
		3 - 3.9%	35	18.9	16.1
		4 - 4.9%	23	18.9	4.1
		5 - 5.9%	19	18.9	0.1
		6 - 6.9%	9	18.9	-9.9
		7 - 7.9%	10	18.9	-8.9
		8 - 8.9%	4	18.9	-14.9
		9 - 9.9%	4	18.9	-14.9
		10%	9	18.9	-9.9
		greater than 10%	12	18.9	-6.9
White	χ^2 (11) = 130.09, *p*<.001	0 - 0.9%	2	18.9	-16.9
56.0%		1 - 1.9%	6	18.9	-12.9
		2 - 2.9%	7	18.9	-11.9
		3 - 3.9%	17	18.9	-1.9
		4 - 4.9%	25	18.9	6.1
		5 - 5.9%	42	18.9	23.1
		6 - 6.9%	16	18.9	-2.9
		7 - 7.9%	19	18.9	0.1
		8 - 8.9%	15	18.9	-3.9
		9 - 9.9%	9	18.9	-9.9
		10%	16	18.9	-2.9
		greater than 10%	53	18.9	34.1

Supplementary Note 4

We conducted a series of separate multi-level models (MLMs) using the *lmer* R package[4] with perceived Asian typicality or perceived status as the predictor variable, Asian subgroup as a moderator (reference = Chinese), and the difference between participants' STEM estimate and population estimate as the dependent variable. Positive numbers for this difference score indicate the overrepresentation of an Asian subgroup, while negative numbers indicate underrepresentation. Each model was conducted twice: once without demographic control variables and once with demographic control variables (i.e., race, age, gender, education, field of current occupation). Given the similarity in methods and measures for Study 1 and Study 2, we meta-analyzed the regression results[5] and report the semipartial correlations[6].

Perceived Asian Typicality

Perceived Asian typicality positively predicted STEM representation, $r_{sp} = 0.07$, *95%CI* [0.004, 0.13], $p = .038$, and this relationship remained significant when control variables were added to the model, r_{sp}=0.07, *95%CI* [0.01, 0.14], $p = .023$. See Table S6 for meta-regression results and Table S7 for individual study results. There were also significant interactions between perceived Asian typicality and the following subgroups: Korean, Filipino, and Vietnamese (see Figure S1). We followed up with simple slopes analyses (see Table S7). In Study 1, the only slopes that were significant were for the Indian and Filipino subgroups; however, with the inclusion of control variables in the model, only the slope for the Indian subgroup remained significant. In Study 2, none of the slopes for the Asian subgroups were significant.

Table S6. Meta-regression analysis for the interaction of perceived Asian typicality and Asian subgroup

*Meta-analysis of semipartial correlations (r_{sp}) between the interaction of perceived Asian typicality and Asian subgroup on STEM – population estimate difference scores for Studies 1 and 2. Semipartial correlations are calculated using the random effects model. The inclusion of control variables in the multi-level regression analysis reduced the overall sample size from N = 784 to N = 767 in Study 1 and N = 197 to N = 196 in Study 2. Note: CI = Confidence Interval, *p<.05*

Effect	Study 1		Study 2		Combined	
	r_{sp}	95% CI	r_{sp}	95% CI	r_{sp}	95% CI
Without Control Variables						
Japanese (vs. Chinese)	0.07	-0.00, 0.14	0.10	-0.05, 0.23	0.07*	0.01, 0.13
Korean (vs. Chinese)	0.04	-0.03, 0.11	0.04	-0.10, 0.18	0.04	-0.02, 0.11
Indian (vs. Chinese)	0.08	0.01, 0.15	0.15	0.01, 0.29	0.09*	0.03, 0.15
Filipino (vs. Chinese)	-0.02	-0.09, 0.05	0.01	-0.13, 0.15	-0.02	-0.08, 0.05
Vietnamese (vs. Chinese)	-0.02	-0.09, 0.05	0.04	-0.10, 0.18	-0.01	-0.07, 0.06
Perceived Asian Typicality	0.06	-0.01, 0.13	0.10	-0.04, 0.24	0.07*	0.00, 0.13
Japanese x Typicality	-0.05	-0.12, 0.02	-0.09	-0.22, 0.05	-0.06	-0.12, 0.01
Korean x Typicality	-0.07	-0.14, 0.00	-0.05	-0.19, 0.09	-0.07*	-0.13, -0.00
Indian x Typicality	0.00	-0.07, 0.07	-0.07	-0.21, 0.07	-0.01	-0.07, 0.05
Filipino x Typicality	-0.09	-0.16, -0.02	-0.11	-0.25, 0.03	-0.09*	-0.15, -0.03
Vietnamese x Typicality	-0.06	-0.13, 0.01	-0.10	-0.24, 0.04	-0.07*	-0.13, -0.00
With Control Variables						
Japanese (vs. Chinese)	0.07	-0.00, 0.14	0.11	-0.03, 0.24	0.08*	0.01, 0.14
Korean (vs. Chinese)	0.05	-0.02, 0.12	0.05	-0.10, 0.19	0.05	-0.20, 0.11
Indian (vs. Chinese)	0.08	0.01, 0.15	0.16	0.02, 0.29	0.10*	0.03, 0.16
Filipino (vs. Chinese)	-0.02	-0.10, 0.05	0.01	-0.12, 0.15	-0.02	-0.08, 0.05
Vietnamese (vs. Chinese)	-0.02	-0.09, 0.06	0.05	-0.10, 0.19	-0.002	-0.07, 0.06
Perceived Asian Typicality	0.06	-0.01, 0.13	0.11	-0.02, 0.25	0.07*	0.01, 0.14
Japanese x Typicality	-0.05	-0.12, 0.02	-0.10	-0.23, 0.04	-0.06	-0.12, 0.00
Korean x Typicality	-0.07	-0.14, -0.00	-0.06	-0.20, 0.08	-0.07	-0.13, -0.01
Indian x Typicality	0.00	-0.07, 0.07	-0.07	-0.21, 0.07	-0.01	-0.08, 0.05
Filipino x Typicality	-0.09	-0.16, -0.02	-0.12	-0.26, 0.02	-0.10*	-0.16, -0.03
Vietnamese x Typicality	-0.07	-0.14, 0.01	-0.11	-0.24, 0.03	-0.07	-0.14, -0.01

Table S7. Perceived Asian typicality and Asian subgroup moderation results and simple slopes analysis.

Results for the moderation analysis and simple slopes analysis testing the interaction between perceived Asian typicality and Asian subgroup on STEM estimate difference score. Control variables include age, race, gender, field, and education.

	Study 1				Study 2			
	Estimate	95% CI	Estimate	95% CI	Estimate	95% CI	Estimate	95% CI
Regression Analysis								
Intercept	-1.12	-3.78, 1.53	-1.57	-7.26, 4.12	-2.90	-8.11, 2.32	-2.96	-8.89, 2.98
Subgroup: Japanese	3.31	-0.06, 6.68	3.40	-0.01, 6.81	4.59	-1.95, 11.13	5.00	-1.63, 11.63
Subgroup: Korean	2.27	-1.17, 5.71	2.30	-1.17, 5.77	1.92	-4.81, 8.65	2.22	-4.57, 9.01
Subgroup: Indian	3.62*	0.49, 6.75	3.71*	0.55, 6.87	6.86*	0.72, 13.01	7.05*	0.78, 13.31
Subgroup: Filipino	-1.12	-4.38, 2.15	-1.09	-4.38, 2.20	0.33	-6.02, 6.67	0.50	-5.94, 6.94
Subgroup: Vietnamese	-0.98	-4.29, 2.34	-0.73	-4.07, 2.61	1.91	-4.52, 8.33	2.25	-4.26, 8.76
Perceived Typicality	0.40	-0.06, 0.85	0.42	-0.04, 0.88	0.66	-0.25, 1.57	0.73	-0.20, 1.66
Japanese x Typicality	-0.43	-1.04, 0.17	0.16	-1.05, 0.17	-0.76	-1.95, 0.44	-0.83	-2.04, 0.38
Korean x Typicality	-0.63*	-1.25, -0.02	-0.63*	-1.26, -0.01	-0.48	-1.71, 0.74	-0.54	-1.78, 0.69
Indian x Typicality	0.02	-0.56, 0.59	0.96	-0.58, 0.59	-0.56	-1.70, 0.58	-0.59	-1.75, 0.57
Filipino x Typicality	-0.81*	-1.42, -0.19	-0.81*	-1.43, -0.20	-0.99	-2.21, 0.22	-1.02	-2.25, 0.21
Vietnamese x Typicality	-0.52	-1.13, 0.09	-0.58	-1.20, 0.04	-0.87	-2.07, 0.33	-0.93	-2.14, 0.28
Simple Slopes Analysis								
Chinese	0.40	-0.06, 0.85	0.42	-0.04, 0.88	0.66	-0.25, 1.57	0.73	-0.19, 1.66
Japanese	-0.04	-0.43, 0.36	-0.02	-0.42, 0.38	-0.10	-0.87, 0.68	-0.10	-0.88, 0.69
Korean	-0.23	-0.65, 0.18	-0.21	-0.63, 0.21	0.18	-0.64, 1.00	0.19	-0.64, 1.02
Indian	0.41*	0.05, 0.77	0.42*	0.06, 0.79	0.10	-0.59, 0.78	0.15	-0.55, 0.84
Filipino	-0.41*	-0.82, -0.00	-0.39	-0.80, 0.02	-0.33	-1.14, 0.47	-0.29	-1.10, 0.53
Vietnamese	-0.12	-0.53, 0.28	-0.16	-0.57, 0.25	-0.21	-0.99, 0.57	-0.19	-0.98, 0.59
Control Variables	No		Yes		No		Yes	

Figure S1. Multi-level model regression results between perceived Asian typicality and Asian subgroup on the STEM - Population estimate difference score

Multi-level model regression results for Study 1 (top) and Study 2 (bottom). Each Asian subgroup is represented by a distinct color. The shaded area surrounding the regression line represents the 95% Confidence Interval.

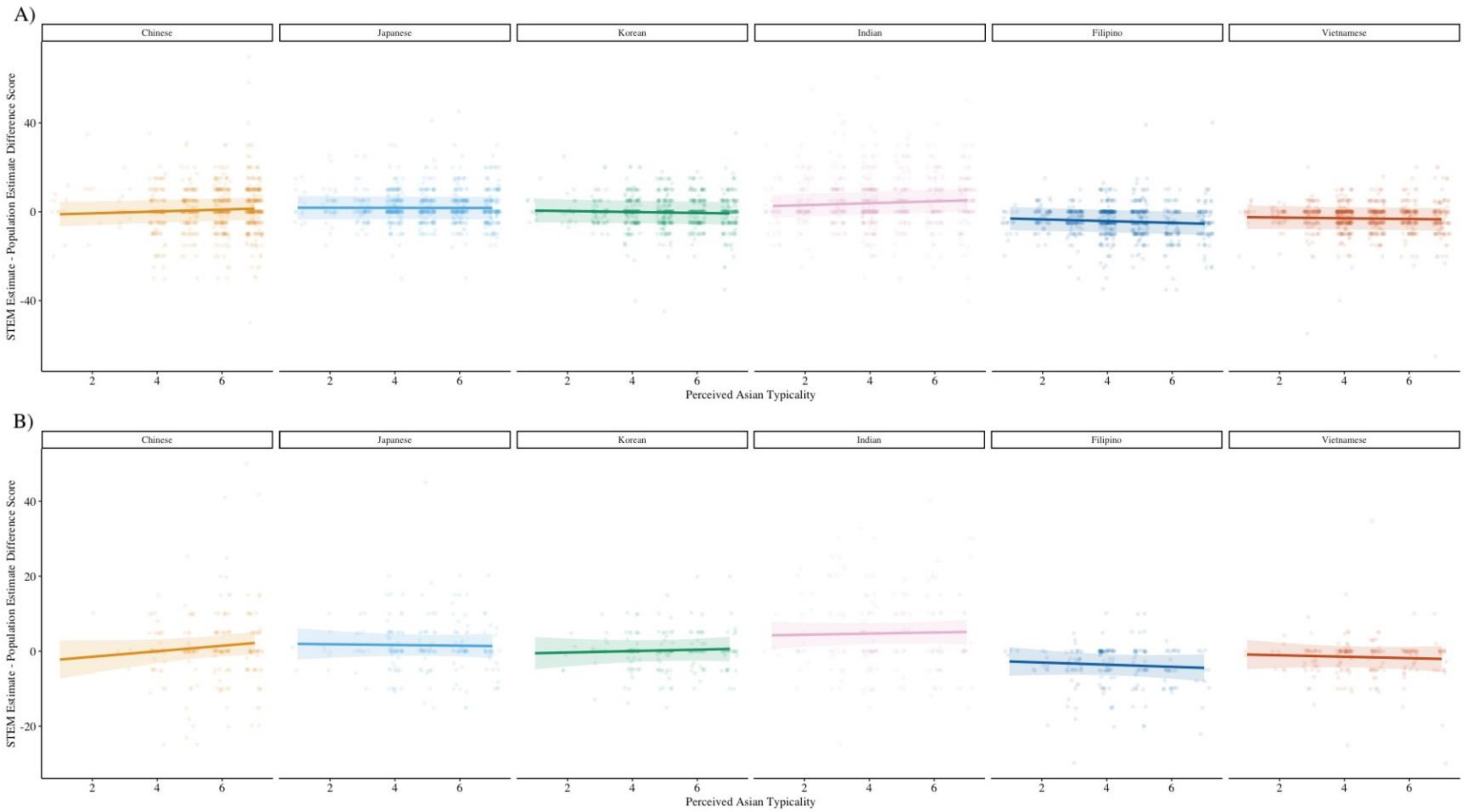

Perceived Status

Perceived status positively predicted STEM representation, $r_{sp} = 0.17$, *95%CI* [0.06, 0.28], $p = .003$, and this relationship remained significant when control variables were added to the model, $r_{sp} = 0.19$, *95%CI* [0.05, 0.32], $p = .007$. See Table S8 for meta-regression results and Table S9 for individual study results. There were also significant interactions between perceived Asian typicality and the following subgroups: Filipino and Vietnamese. We followed up with simple slopes analyses (see Table S9 and Figure S2). In Study 1, the slopes for the Chinese, Japanese, Korean, and Indian subgroups were positive and significant while the slopes for the Filipino and Vietnamese subgroups were not significant. However, in Study 2, the slopes for only the Chinese and Indian subgroups were positive and significant. The slope for the Korean subgroup became significant when control variables were included in the model.

Table S8. Meta-regression analysis for the interaction of perceived status and Asian subgroup

*Meta-analysis of semipartial correlations (r_{sp}) between the interaction of perceived status and Asian subgroup on STEM – population estimate difference scores for Studies 1 and 2. Semipartial correlations are calculated using the random effects model. The inclusion of control variables in the multi-level regression analysis reduced the overall sample size from N = 784 to N = 767 in Study 1 and N = 197 to N = 196 in Study 2. Note: CI = Confidence Interval, *p<.05*

Effect	Study 1		Study 2		Combined	
	r_{sp}	95% CI	r_{sp}	95% CI	r_{sp}	95% CI
Without Control Variables						
Japanese (vs. Chinese)	0.01	-0.06, 0.08	0.18	0.04, 0.31	0.08	-0.08, 0.24
Korean (vs. Chinese)	0.01	-0.06, 0.08	0.06	-0.08, 0.20	0.02	-0.04, 0.08
Indian (vs. Chinese)	0.01	-0.06, 0.08	0.12	-0.02, 0.25	0.05	-0.05, 0.15
Filipino (vs. Chinese)	-0.02	-0.09, 0.05	0.03	-0.11, 0.17	-0.01	-0.07, 0.05
Vietnamese (vs. Chinese)	-0.00	-0.07, 0.07	0.10	-0.04, 0.23	0.03	-0.06, 0.12
Perceived Status	0.13	0.06, 0.20	0.25	0.11, 0.37	0.17*	0.06, 0.28
Japanese x Status	0.00	-0.07, 0.07	-0.18	-0.31, -0.04	-0.08	-0.26, 0.10
Korean x Status	-0.03	-0.10, 0.04	-0.08	-0.22, 0.06	-0.04	-0.10, 0.03
Indian x Status	0.06	-0.01, 0.13	-0.04	-0.18, 0.10	0.03	-0.07, 0.13
Filipino x Status	-0.07	-0.14, -0.00	-0.10	-0.24, 0.04	-0.08*	-0.14, -0.02
Vietnamese x Status	-0.06	-0.13, 0.01	-0.14	-0.27, 0.00	-0.07*	-0.14, -0.01
With Control Variables						
Japanese (vs. Chinese)	0.01	-0.07, 0.08	0.19	0.05, 0.32	0.09	-0.09, 0.26
Korean (vs. Chinese)	0.002	-0.07, 0.07	0.07	-0.07, 0.21	0.02	-0.05, 0.08
Indian (vs. Chinese)	0.01	-0.06, 0.08	0.13	-0.01, 027	0.05	-0.06, 0.17
Filipino (vs. Chinese)	-0.02	-0.09, 0.05	0.03	-0.11, 0.17	-0.01	-0.07, 0.05
Vietnamese (vs. Chinese)	-0.004	-0.08, 0.07	0.10	-0.04, 0.24	0.03	-0.07, 0.13
Perceived Status	0.13	0.06, 0.20	0.27	0.14, 0.40	0.19*	0.05, 0.32
Japanese x Status	0.01	-0.06, 0.08	-0.19	-0.33, -0.06	-0.08	-0.27, 0.11
Korean x Status	-0.02	-0.09, 0.05	-0.09	-0.23, 0.05	-0.03	-0.10, 0.03
Indian x Status	0.07	-0.00, 0.14	-0.05	-0.19, 0.09	0.03	-0.09, 0.14
Filipino x Status	-0.07	-0.14, 0.00	-0.11	-0.24, 0.03	-0.08*	-0.14, -0.01
Vietnamese x Status	-0.06	-0.13, 0.01	-0.14	-0.28, -0.00	-0.08*	-0.15, -0.01

Table S9. Perceived status and Asian subgroup moderation results and simple slopes analysis.

Results for the moderation analysis and simple slopes analysis testing the interaction between perceived status and Asian subgroup on STEM estimate difference score. Control variables include age, race, gender, field, and education.

	Study 1				Study 2			
	Estimate	95% CI	Estimate	95% CI	Estimate	95% CI	Estimate	95% CI
Regression Analysis								
Intercept	-3.94*	-6.62, -1.26	-3.73*	-9.35, 1.89	-8.03*	-12.96, -3.11	-7.83	-13.34, -2.33
Subgroup: Japanese	0.64	-3.26, 4.54	0.33	-3.61, 4.28	10.38*	2.57, 18.19	10.68*	2.82, 18.53
Subgroup: Korean	0.5	-3.21, 4.20	0.09	-3.66, 3.84	3.25	-3.76, 10.26	3.41	-3.61, 10.43
Subgroup: Indian	0.54	-2.90, 3.99	0.35	-3.13, 3.83	5.76	-0.79, 12.30	6.07	-0.48, 12.63
Subgroup: Filipino	-0.91	-4.30, 2.47	-1.06	-4.47, 2.36	1.37	-4.94, 7.67	1.40	-4.92, 7.73
Subgroup: Vietnamese	-0.18	-3.55, 3.18	-0.21	-3.61, 3.18	4.45	-1.76, 10.66	4.64	-1.59, 10.87
Perceived Status	0.73*	0.35, 1.10	0.74*	0.36, 1.12	1.30*	0.59, 2.01	1.40*	0.69, 2.12
Japanese x Status	0.02	-0.52, 0.57	0.06	-0.48, 0.61	-1.46*	-2.54, -0.37	-1.50*	-2.59, -0.42
Korean x Status	-0.21	-0.75, 0.32	-0.16	-0.69, 0.38	-0.61	-1.62, 0.40	-0.64	-1.65, 0.37
Indian x Status	0.48	-0.02, 0.98	0.51*	0.00, 1.01	-0.30	-1.24, 0.65	-0.34	-1.29, 0.61
Filipino x Status	-0.47*	-1.11, -0.04	-0.55*	-1.09, -0.01	-0.79	-1.80, 0.23	-0.77	-1.79, 0.24
Vietnamese x Status	-0.45	-0.98, 0.08	-0.46	-0.99, 0.07	-0.99*	-1.98, -0.01	-1.00	-1.99, -0.02
Simple Slopes Analysis								
Chinese	0.73*	0.35, 1.10	0.74*	0.26, 1.12	1.30	0.59, 2.01	1.40	0.69, 2.12
Japanese	0.75*	0.36, 1.14	0.80*	0.41, 1.20	-0.15	-0.97, 0.66	-0.10	-0.93, 0.72
Korean	0.51*	0.14, 0.89	0.58*	0.20, 0.96	0.69	-0.02, 1.41	0.76	0.03, 1.48
Indian	1.21*	0.88, 1.53	1.25*	0.92, 1.58	1.01	0.38, 1.64	1.06	0.42, 1.69
Filipino	0.16	-0.23, 0.54	0.19	-0.19, 0.58	0.51	-0.21, 1.24	0.63	-0.10, 1.36
Vietnamese	0.28	-0.09, 0.64	0.28	-0.09, 0.65	0.31	-0.38, 0.99	0.40	-0.29, 1.09
Control Variables	No		Yes		No		Yes	

Figure S2. Multi-level model regression results between perceived status and Asian subgroup on the STEM - Population estimate difference score

Multi-level model regression results for Study 1 (top) and Study 2 (bottom). Each Asian subgroup is represented by a distinct color. The shaded area surrounding the regression line represents the 95% Confidence Interval.

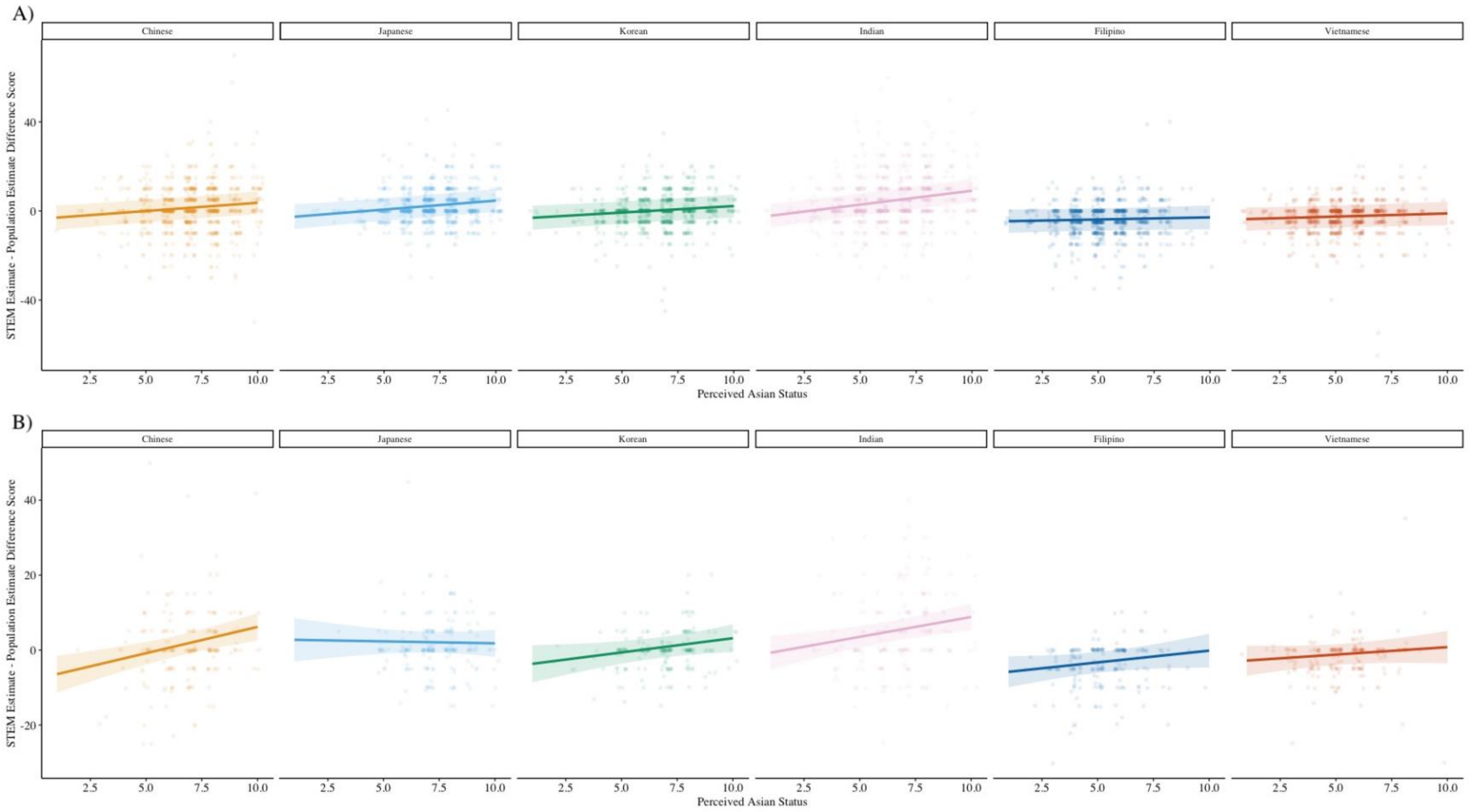

Supplementary References

1. Epley, N., & Gilovich, T. (2001). Putting adjustment back in the anchoring and adjustment heuristic: Differential processing of self-generated and experimenter-provided anchors. Psychological Science, 12(5), 391–396. https://doi.org/10.1111/1467-9280.00372
2. Tversky, A., & Kahneman, D. (1974). Judgment under uncertainty: Heuristics and biases. Science, 185(4157), 1124–1131. https://doi.org/10.1126/science.185.4157.1124
3. U.S. Census Bureau. (2023). *CPS Annual Social and Economic (March) Supplement (2023 MAR).* Retrieved from https://data.census.gov/app/mdat/CPSASEC2023/vars.
4. Bates D, Mächler M, Bolker B, Walker S (2015). "Fitting Linear Mixed-Effects Models Using lme4." *Journal of Statistical Software*, **67**(1), 1–48. doi:10.18637/jss.v067.i01.
5. Goh, J. X., Hall, J. A., & Rosenthal, R. (2016). Mini meta-analysis of your own studies: Some arguments on why and a primer on how. *Social and Personality Psychology Compass*, *10*(10), 535-549. https://doi.org/10.1111/spc3.12267
6. Aloe, A. M., & Becker, B. J. (2012). An effect size for regression predictors in meta-analysis. *Journal of Educational and Behavioral Statistics*, *37*(2), 278-297. https://doi.org/10.3102/10769986103969
