# Supplementary material for: The misperception of Asian subgroup representation in STEM
Source: Commun Psychol. 2026 Jan 3;4:21. doi: 10.1038/s44271-025-00389-1 (PMC12873391; doi:10.1038/s44271-025-00389-1)
Supplement: Supplementary file 3 — Reporting Summary [file 44271_2025_389_MOESM3_ESM.pdf]

nature portfolio

Corresponding author(s): COMMSPSYCHOL-25-0503-T

Last updated by author(s): 2025_10_10

Reporting Summary

Nature Portfolio wishes to improve the reproducibility of the work that we publish. This form provides structure for consistency and transparency in reporting. For further information on Nature Portfolio policies, see our Editorial Policies and the Editorial Policy Checklist.

Statistics

For all statistical analyses, confirm that the following items are present in the figure legend, table legend, main text, or Methods section.

n/a	Confirmed	
☐	☒	The exact sample size (n) for each experimental group/condition, given as a discrete number and unit of measurement
☐	☒	A statement on whether measurements were taken from distinct samples or whether the same sample was measured repeatedly
☐	☒	The statistical test(s) used AND whether they are one- or two-sided<br>*Only common tests should be described solely by name; describe more complex techniques in the Methods section.*
☐	☒	A description of all covariates tested
☐	☒	A description of any assumptions or corrections, such as tests of normality and adjustment for multiple comparisons
☐	☒	A full description of the statistical parameters including central tendency (e.g. means) or other basic estimates (e.g. regression coefficient) AND variation (e.g. standard deviation) or associated estimates of uncertainty (e.g. confidence intervals)
☐	☒	For null hypothesis testing, the test statistic (e.g. F, t, r) with confidence intervals, effect sizes, degrees of freedom and P value noted<br>*Give P values as exact values whenever suitable.*
☒	☐	For Bayesian analysis, information on the choice of priors and Markov chain Monte Carlo settings
☐	☒	For hierarchical and complex designs, identification of the appropriate level for tests and full reporting of outcomes
☐	☒	Estimates of effect sizes (e.g. Cohen's d, Pearson's r), indicating how they were calculated

Our web collection on statistics for biologists contains articles on many of the points above.

Software and code

Policy information about availability of computer code

Data collection	Data was collected using Qualtrics software and is available on the Open Science Framework: https://osf.io/g7z8x/?view_only=07b45c2d46a84888af3fd210731e6d41
Data analysis	Data was analyzed using a combination of SPSS and R - both data analysis scripts are available on the Open Science Framework: https://osf.io/g7z8x/?view_only=07b45c2d46a84888af3fd210731e6d41

For manuscripts utilizing custom algorithms or software that are central to the research but not yet described in published literature, software must be made available to editors and reviewers. We strongly encourage code deposition in a community repository (e.g. GitHub). See the Nature Portfolio guidelines for submitting code & software for further information.

Data

Policy information about availability of data

All manuscripts must include a data availability statement. This statement should provide the following information, where applicable:

- Accession codes, unique identifiers, or web links for publicly available datasets
- A description of any restrictions on data availability
- For clinical datasets or third party data, please ensure that the statement adheres to our policy

Data files, syntax, and materials are available on the Open Science Framework (https://osf.io/g7z8x/?view_only=07b45c2d46a84888af3fd210731e6d41).

Research involving human participants, their data, or biological material

Policy information about studies with human participants or human data. See also policy information about sex, gender (identity/presentation), and sexual orientation and race, ethnicity and racism.

Reporting on sex and gender	We asked participants to provide their gender identity in our demographics form (i.e., they were asked to select their gender identity from a list of provided options). We did not perform any analyses comparing gender. We did control for reported gender identity in our multi-level analyses determining if perceived status and Asian typicality separately predicted STEM estimations. Our results suggest that our findings generalize to both men and women. The main manuscript has a detailed table of the demographic statistics for each of our study samples: Study 1 (men = 369, women = 383, non-binary = 14), Study 2 (men = 106, women = 86, non-binary = 5), Study 3 (men = 189, women = 255, non-binary = 7), and Study 4 (men = 341, women = 354, non-binary = 9).
Reporting on race, ethnicity, or other socially relevant groupings	We asked participants their racial identity in our demographics form (i.e., they were asked to select their racial identity from a list of provided options). In Study 1, we tried to get at least 200 participants who identified as White, Black, Asian, and Latine to compare if STEM estimations differed depending on race. Due to Prolific racial demographic item being slightly different than our demographic form (i.e., Prolific did not have Pacific Islander as a racial identity option while we did), we had a sample of White (n = 190), Black (n= 180), Asian (n = 192), Latine (n = 134), and Pacific Islander (n = 71). In Study 1, we conducted a 5(participant race: White, Black, Asian, Latine, Pacific Islander) x 6 (Asian subgroup: Chinese, Japanese, Korean, Indian, Filipino, Vietnamese) mixed-model ANOVA to determine if Asian participants were more accurate in their STEM estimations than the other racial groups. We found that while Asian participants were slightly more accurate in estimating Indian and Japanese subgroup representation in STEM than other racial groups, Asian participants still misperceived STEM representation for each Asian subgroup just like the other racial groups. We additionally did control for reported racial identity in our multi-level analyses determining if perceived status and Asian typicality separately predicted STEM estimations. Our results suggest that our findings generalize to all racial groups.
Population characteristics	See above
Recruitment	Participants were recruited using a convenience sample from Prolific.
Ethics oversight	The materials and methods for Studies 1-3 were reviewed and approved by the Institutional Review Board at Yale University, while the Institutional Review Board at Northwestern University approved the materials and methods for Study 4.

Note that full information on the approval of the study protocol must also be provided in the manuscript.

Field-specific reporting

Please select the one below that is the best fit for your research. If you are not sure, read the appropriate sections before making your selection.

☐ Life sciences ☒ Behavioural & social sciences ☐ Ecological, evolutionary & environmental sciences

For a reference copy of the document with all sections, see nature.com/documents/nr-reporting-summary-flat.pdf

Behavioural & social sciences study design

All studies must disclose on these points even when the disclosure is negative.

Study description	All four studies are quantitative.
Research sample	The research sample for all four studies were U.S. adults. Study 1: N = 784 Prolific participants; Mage = 37.72, SDage = 12.70; 47% men, 49% women; 24% White, 23% Black, 24% Asian, 17% Latinx, 9% Pacific Islander. Study 2: N = 197 Prolific participants; Mage = 43.97, SDage = 13.50; 54% men, 44% women; 60% White, 19% Black, 12% Asian, 3% Latinx, 6% Pacific Islander. Study 3: N = 451 Prolific participants; Mage = 37.88, SDage = 11.69; 42% men, 57% women; 75% White, 6% Black,10% Asian, 4% Latinx, 6% Pacific Islander. Study 4 (nationally representative sample): N =708 Prolific participants; Mage = 46.54, SDage = 25.07; 48% men, 50% women; 62% White,13% Black, 6% Asian, 6% Latinx, 7% Pacific Islander.
Sampling strategy	We used an online convenience sample from Prolific for all four studies. For each study, we aimed to recruit at least n = 200 participants per condition or unit of between-subjects analysis (e.g., participant race).
Data collection	Participants completed the survey online using Qualtrics.
Timing	Study 1: 2024_02_12<br>Study 2: 2024_03_04<br>Study 3: 2024_06_03<br>Study 4: 2025_01_06
Data exclusions	We only excluded participants from Study 1 and 2 (details are in Table S1 of the supplement). In Study 1, we excluded n = 15 participants leaving N = 784 participants and in Study 2, we excluded n = 32 participants leaving N = 197. In both studies, our exclusion criteria was that participants' STEM estimations for each Asian subgroup had to total between 90 and 110. We gave explicit instructions that participants' total STEM estimations had to equal 100. We included the +/- 10 as a buffer. Additionally, in Study 2,

	we wanted to recruit participants who indicated they had a Masters or Ph.D. in a STEM field. Although, we used a pre-screening item on Prolific, we excluded participants who did not report they had a Masters or a Ph.D.
Non-participation	N/A
Randomization	In Studies 1 and 2, there was not a between-subjects factor. In Study 3, participants were randomly assigned to either the open-ended condition or the close-ended condition. In Study 4, participants were randomly assigned to either the control condition or the intervention condition. Qualtrics randomly assigned participants to the conditions in both studies 3 and 4.

Reporting for specific materials, systems and methods

We require information from authors about some types of materials, experimental systems and methods used in many studies. Here, indicate whether each material, system or method listed is relevant to your study. If you are not sure if a list item applies to your research, read the appropriate section before selecting a response.

Materials & experimental systems

n/a	Involved in the study
☒	☐ Antibodies
☒	☐ Eukaryotic cell lines
☒	☐ Palaeontology and archaeology
☒	☐ Animals and other organisms
☒	☐ Clinical data
☒	☐ Dual use research of concern
☒	☐ Plants

Methods

n/a	Involved in the study
☒	☐ ChIP-seq
☒	☐ Flow cytometry
☒	☐ MRI-based neuroimaging

Plants

Seed stocks	*Report on the source of all seed stocks or other plant material used. If applicable, state the seed stock centre and catalogue number. If plant specimens were collected from the field, describe the collection location, date and sampling procedures.*
Novel plant genotypes	*Describe the methods by which all novel plant genotypes were produced. This includes those generated by transgenic approaches, gene editing, chemical/radiation-based mutagenesis and hybridization. For transgenic lines, describe the transformation method, the number of independent lines analyzed and the generation upon which experiments were performed. For gene-edited lines, describe the editor used, the endogenous sequence targeted for editing, the targeting guide RNA sequence (if applicable) and how the editor was applied.*
Authentication	*Describe any authentication procedures for each seed stock used or novel genotype generated. Describe any experiments used to assess the effect of a mutation and, where applicable, how potential secondary effects (e.g. second site T-DNA insertions, mosiacism, off-target gene editing) were examined.*
